# The effectiveness of proactive telephone support provided to breastfeeding mothers of preterm infants: study protocol for a randomized controlled trial

**DOI:** 10.1186/1471-2431-13-73

**Published:** 2013-05-10

**Authors:** Jenny Ericson, Mats Eriksson, Lena Hellström-Westas, Lars Hagberg, Pat Hoddinott, Renée Flacking

**Affiliations:** 1 Department of Women’s and Children’s Health, Uppsala University, Uppsala, Sweden; 2 Centre for Clinical Research Dalarna, Nissersväg 3, Falun, S-79182, Sweden; 3 Department of Pediatrics, Falu Hospital, Falun, Sweden; 4 School of Health and Medical Sciences, Örebro University, Örebro, Sweden; 5 Centre for Health Care Sciences, Örebro University Hospital, Örebro, Sweden; 6 Nursing, Midwifery and Allied Health Professionals Research Unit, University of Stirling, Stirling, Scotland; 7 School of Health and Social Studies, Dalarna University, Falun, Sweden; 8 Maternal and Infant Nutrition and Nurture Unit (MAINN), School of Health, University of Central Lancashire, Preston, Lancashire, UK

**Keywords:** Breastfeeding, Mothers, Neonatal care, Preterm infant, Support, Telephone

## Abstract

**Background:**

Although breast milk has numerous benefits for infants’ development, with greater effects in those born preterm (at < 37 gestational weeks), mothers of preterm infants have shorter breastfeeding duration than mothers of term infants. One of the explanations proposed is the difficulties in the transition from a Neonatal Intensive Care Unit (NICU) to the home environment. A person-centred proactive telephone support intervention after discharge from NICU is expected to promote mothers’ sense of trust in their own capacity and thereby facilitate breastfeeding.

**Methods/design:**

A multicentre randomized controlled trial has been designed to evaluate the effectiveness and cost-effectiveness of person-centred proactive telephone support on breastfeeding outcomes for mothers of preterm infants. Participating mothers will be randomized to either an intervention group or control group. In the intervention group person-centred proactive telephone support will be provided, in which the support team phones the mother daily for up to 14 days after hospital discharge. In the control group, mothers are offered a person-centred reactive support where mothers can phone the breastfeeding support team up to day 14 after hospital discharge. The intervention group will also be offered the same reactive telephone support as the control group. A stratified block randomization will be used; group allocation will be by high or low socioeconomic status and by NICU. Recruitment will be performed continuously until 1116 mothers (I: 558 C: 558) have been included. Primary outcome: proportion of mothers exclusively breastfeeding at eight weeks after discharge. Secondary outcomes: proportion of breastfeeding (exclusive, partial, none and method of feeding), mothers satisfaction with breastfeeding, attachment, stress and quality of life in mothers/partners at eight weeks after hospital discharge and at six months postnatal age. Data will be collected by researchers blind to group allocation for the primary outcome. A qualitative evaluation of experiences of receiving/providing the intervention will also be undertaken with mothers and staff.

**Discussion:**

This paper presents the rationale, study design and protocol for a RCT providing person-centred proactive telephone support to mothers of preterm infants. Furthermore, with a health economic evaluation, the cost-effectiveness of the intervention will be assessed.

**Trial registration:**

NCT01806480

## Background

In most industrialized countries breastfeeding rates are far from the World Health Organization’s (WHO) recommendation of exclusive breastfeeding, with no other fluids or solids, for 6 months after birth [1] and hence efforts have been made to increase breastfeeding rates. A systematic review of 52 trials from 21 countries has demonstrated that lay support and additional professional support were the only effective interventions prolonging breastfeeding [2]. Telephone support interventions to increase breastfeeding duration have shown promise [3]. However, it has been suggested that the reason why breastfeeding telephone support interventions sometimes fail is that care is reactive rather than proactive [3].

A qualitative meta-synthesis of women’s experiences shows the importance of providing person-centred care in supporting breastfeeding [4]. The synthesis, which is derived mostly from face-to-face interactions, suggests that authentic presence and a facilitative approach, which involve supportive care and a trusting relationship, are experienced as helpful for women who want to breastfeed. McCormack and colleagues [5,6] have defined person-centred care as a model of care, which includes components such as: building mutual trust and understanding; treating the person as an individual; respecting the rights of the person, sharing decision-making, providing holistic care and developing therapeutic relationships. Furthermore, the care provider should engage with and have a sympathetic presence with the person [6]. It is suggested that outcomes of effective person-centred care are increased satisfaction with care, involvement in care, and a feeling of well-being [6].

Breastfeeding has been shown to be highly beneficial for nutritional, immunological, and cognitive development. Investments in service and strategies to enhance and maintain breastfeeding frequency and exclusivity can be cost-effective and increase the quality of life in infants by reducing acute and chronic diseases [7,8].

Exclusive breastfeeding has advantages compared to partial breastfeeding in decreasing the risk of diarrhoea, respiratory illness, otitis media, necrotizing enterocolitis (NEC), atopic dermatitis and type 1 diabetes [9]. In preterm infants (born < 37 gestational weeks) breastfeeding advantages are even more pronounced [10,11]. Vohr et al. [12] studied the effects of breast milk on infant development measured by Bayley Scales and showed a significant independent association with outcomes. For every 10 mL/kg increase of breast milk intake per day the Mental Developmental Index increased by 0.53 points, the Psychomotor Development Index by 0.63 points and finally the Behavior Rating Scale by 0.82 points. Also, the likelihood of re-hospitalization decreased by 6%; on all results after adjusting for socioeconomic status (SES), maternal age, marital status and ethnicity.

Breastfeeding rates varies internationally; from the Nordic countries where almost all mothers initiate breastfeeding to countries such as France and Ireland with less than a 70% initiation rate [13]. Breastfeeding rates among preterm infants are much lower than in term infants [14], with a wide variation observed in the preterm population [15-18]. Furthermore, studies of preterm infants show that the proportion of very preterm (< 32 gestational weeks) infants who are exclusively breastfed at 2, 4 and 6 months corrected age is lower than among moderately preterm infants [17,19].

A number of studies show a significant relationship between socioeconomic status (SES) and breastfeeding duration; disadvantaged mothers discontinue breastfeeding much earlier than mothers with more advantageous positions [14,20-22]. The best proxy for SES is suggested to be educational level [23]. A previous Swedish study reported that 87% of mothers with a higher education were breastfeeding their preterm infant at two months of postnatal age, compared to 80% of those with an upper secondary school, and 58% of those with compulsory school or less [21]. Unpublished data from a study by Wallin et al. [19] suggests that SES seems to have an even greater impact on exclusive breastfeeding; 68% of the mothers with a higher education breastfed exclusively at two months compared to 47% of the mothers with a lower educational level.

Compared to term infants, preterm infants are immature in their development and cannot be fully breastfed immediately after birth. Instead, mothers and infants experience a transition period from tube feeding to breastfeeding, in which mothers cannot take full responsibility for their infants’ nutrition and survival in the same way as parents of healthy infants born at term can [24]. Compared to mothers of term infants, mothers of preterm infants spend longer being a mother in a public hospital environment, in which they may become dependent on the benevolence and support of the staff [25-27]. The transition from a medical setting to a home environment can be difficult for the mothers, because of unsolved grief, imprints of institutional authority and feelings of guilt and shame [24,28]. Improved support to families after discharge from the NICUs has hence been suggested as crucial for parental role attainment and to help families with the transition to the home environment [29-32].

This person-centered proactive telephone support intervention aims to increase exclusive breastfeeding rates, assess the cost-effectiveness of the intervention, improve maternal satisfaction, increase parental attachment, wellbeing and reduce parental stress.

## Methods/design

### Aim and hypotheses

This is a multi-centre randomized controlled trial (RCT), blinded for research team for primary outcome measurement. The primary aim of the RCT is to evaluate the effectiveness of proactive person-centred telephone support provided to breastfeeding mothers of preterm infants for up to 14 days after hospital discharge from NICUs on exclusive breastfeeding. We hypothesize that proactive (health service initiated) telephone support offered to breastfeeding (exclusive or partial) mothers of preterm infants after hospital discharge is more effective than reactive (mother initiated, and defined as usual care) telephone support at increasing the proportion of mothers who are exclusively breastfeeding 8 weeks after discharge.

The secondary aim is to evaluate the effectiveness and cost-effectiveness of proactive telephone support on breastfeeding (exclusive, partial, none and method of feeding), mothers satisfaction with breastfeeding, attachment, parental stress and quality of life in mothers/partners at 8 weeks after hospital discharge and at six months postnatal age. We hypothesize that breastfeeding, maternal satisfaction with breastfeeding and attachment, parental stress, and quality in life will be improved in mothers and partners who receive proactive telephone support. In addition, a qualitative evaluation will be performed, by interviewing mothers and staff on their experiences of receiving and delivering proactive person-centred telephone support respectively.

This intervention has been piloted by Professor Hoddinott and colleagues on a postnatal ward in Scotland [33,34]. The implementation, experiences, process evaluation and results from that pilot have provided this planned RCT with important knowledge, crucial for the design. For example, the pilot study reported a 0.23 effect size in breastfeeding 6–8 weeks and that the median telephone call time was 5 minutes.

### Definitions

For the purpose of this trial, we have used the following definitions. Breastfeeding refers to a mother providing any breast milk to her infant, regardless of method. Exclusive breastfeeding refers to only breast milk with no other fluids or solids except for medications and vitamins, given to the infant in the previous 24 hours. Partial breastfeeding refers to breast milk and infant formula, given to the infant in the previous 24 hours.

Person-centred care is defined as building mutual trust and understanding between caregiver and person; treating the person as an individual; respecting the rights of the person, sharing decision-making, providing holistic care and developing therapeutic relationships. The care provider should also engage with and have a sympathetic presence with the person.

### Study sample

The study setting will be four general or referral NICUs level IIIa or IIIb [35], geographically spread over Sweden. Eligible participants for randomization are mothers with preterm infants (< 37 gestational weeks), admitted to one of the four selected NICUs for at least 48 hours and who breastfeed or express breast milk. Exclusion criteria: serious maternal medical or psychiatric problems at discharge; language problems that cannot be resolved; the infant is transferred to another hospital/unit after discharge; infants that are terminally ill.

### Sample size calculation

A priori power analysis has been calculated to determine an adequate sample size for the study. This is a study of independent cases and controls with 1 control per case. Prior data indicate that the exclusive breastfeeding rate at two months of corrected age in preterm infants is 0.53. If the true exclusive breastfeeding rate for intervention mothers is 0.615 (effect size 0.085), we will need to study 531 intervention mothers and 531 control mothers to be able to reject the null hypothesis that the exclusive breastfeeding rates for intervention and control mothers are equal with a probability (power) of 0.8, using the Chi-square test. The Type I error probability associated with this test of the null hypothesis is 0.05. We estimate that the drop-out rate will be 5%, therefore requiring an additional 54 mothers. In total we need a sample size of at least 1116 mothers (I: 558, C: 558). The trial is also powered for the subgroup, low SES mothers. It is estimated that the study period will be 18 months.

### Recruitment of the breastfeeding support team

A breastfeeding support team (BST) will be set up in each of the four NICUs (7 staff members/each unit). In the selection of BST members, personal qualities (e.g. warm personality, organizational skills) and willingness to be part of the team are important. The selection will be conducted differently in the NICUs depending on the NICU’s organization, culture in constituting ‘groups’ and feasibility.

### Recruitment procedure for participants

The BST members at each NICU will inform eligible mothers and their partners, verbally and in writing, about the study, 1–2 weeks prior to the infant’s potential discharge. Mothers who consent to participate in the study will be randomly allocated immediately after hospital discharge (within 24 h) to one of two groups; intervention (I), or control group (C).

### Randomization process

**Figure 1:**
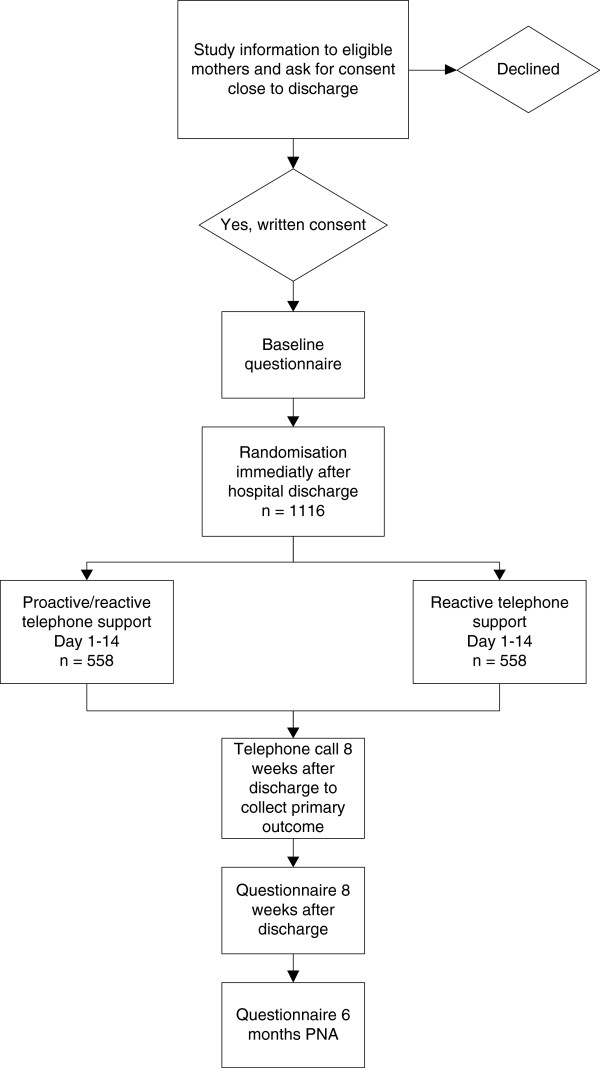
Flowchart on the progress of the study.

Mothers who meet the inclusion criteria and who provide consent will be randomized to either the proactive (I) or reactive (C) telephone support group. Each mother will be assigned an identification code when they consent to participate, which will be used at randomization and on all questionnaires. The randomization is done by the BST immediately after discharge (within 24 h) by an automated and secure web-based system administrated independent of the research team. A stratified block randomization will be used, with blocks of 25 high SES and 25 low SES mothers at each participating NICU. The randomization takes place after the BST have collected baseline birth, feeding and socio-demographic data so that the BST or mothers will not be biased by knowing the group assignment. The mothers will be informed of their randomization group immediately following randomization by a telephone call or text message. The study outline is presented in Figure 1.

### Intervention

The intervention in this study is proactive telephone support initiated by the BST based at the NICU from which the infant is discharged. Daily phone calls from a member of the BST to the mother will be performed from day 1 until day 14 after discharge. In addition, the mother has the option to call someone in the BST during the same period (reactive telephone support). The telephone support will be conducted with a person-centered approach, as defined earlier and will aim to provide continuity of care. Thus, the mother is enabled to talk about whatever feels important to her and establish a trusting relationship with BST members.

### Control group

The control group will be offered the opportunity for person-centred reactive telephone support initiated by the mother who can phone the support team from day 1 after discharge until day 14 after discharge, 08.00-16.00 every day. Each NICU will set up a specific telephone number for their telephone support, and schedule BST members to be available. The same level of person-centeredness will be provided for the mothers in the control group (reactive) as mothers in the intervention group (proactive).

### Study protocol

The BST members at each site will be educated in a two-day course prior study start provided by the first and last author. The course includes theoretical sessions on becoming a mother in a NICU environment, person centered care and breastfeeding physiology. It also includes a practical session on telephone support (including in how to handle cases when triage to other pathways are needed), and a seminar on research methodology with focus on conducting a RCT. The BST members will receive in-depth information about the study and instructions to procedures for handling informed consent, protocols, log books, baseline questionnaire and telephone systems.

Each unit will keep a Log-book, in which the BST will record data on all infants admitted to the NICU, admission date, gestation week, eligibility for inclusion, date assessed for eligibility, reason for exclusion and whether mothers that decline participation have been asked to voluntarily answer some baseline characteristic questions (i.e. age, parity, gestational age, delivery and maternal educational level). Data on mothers/partners and infants participating in the trial (i.e. name, phone number, address, identification code, and infant’s date of birth) will be recorded in the Log-book prior to hospital discharge. In addition, if any mothers drop out of the trial between informed consent and hospital discharge, the reasons will be recorded. Established data protocols on demographics, infant health and breastfeeding are filled in by a BST member at inclusion and at discharge. All information collected prior hospital discharge, will be forwarded to the researcher in charge, together with data on date of discharge. After hospital discharge, randomization will generate the group allocation which will be entered in to the log book. Only the BST in each unit will know the allocated group for each mother. The identification code will be used to identify all distributed questionnaires and link data to each trial participant. Project coordination and data collection at eight weeks after discharge and six months of infant’s postnatal age will be performed by the first author blind to randomization for the primary outcome. University data protection and quality assurance procedures will be followed. For quality control of data entry, another member of the research team will do a random check of data entry quality in 10% of sample cases.

### Baseline socio-demographic and birth data collection

Data collection for participating mothers and partners include: educational level, parity, mode of delivery, ethnicity, and smoking habits. Data collection for participating infants include: gender, single or multiple birth, gestational age (GA) at birth, weight at birth, days on ventilator/ Continuous Positive Airway Pressure (CPAP), length of hospital stay, GA and weight at discharge, neonatal sequelae at discharge, breast milk (exclusive, partial, formula milk) and method (breastfeeding, bottle, cup) at discharge. Table 1 details the time points when baseline data will be collected.

**Table 1:** Time schedule for obtaining data on characteristics

**Baseline data**	**All infants admitted at the NICU**	**Trial participants at enrolment**	**Trial participants at discharge**
Date of birth	•	•	
Sex (infant)	•	•	
Gestational age	•	•	
Age (mothers/partners)		•	
Parity		•	
Birth weight		•	
Way of delivery		•	
Single/multiple birth		•	
Educational level (mother)		•	
Ethnicity (mother/partner)		•	
Weight at discharge			•
Date of discharge			•
Days ‘on leave’ before discharge*			•
Infant’s health			•
Parent’s health			•

*In Sweden, parents are sometimes enabled to take their infants home (hours or days), although still admitted to the NICU.

### Primary outcome

Data on feeding status at eight weeks after discharge will be obtained through a telephone call in which mothers are asked if they give their infant breast milk (i.e. exclusive, partial, none), the method of feeding (i.e. breast, bottle, cup, tube) and infant’s weight. If the mothers have ceased breast milk feeding, they are asked at what time they ceased. The person who makes the phone call will be blinded for study group.

### Secondary outcomes

Table 2 details the time points when the following outcomes will be collected:

Parental stress in mothers and partners will be measured through the Swedish Parenthood Stress Questionnaire (SPSQ) [36], an adapted version of the Parental Stress Index [37]. It measures perceived stress in parenting in five dimensions (incompetence, role restriction, social isolation, spouse relationship and health problems) and has 34 items.

Quality of life in mothers and partners will be measured through the Short-Form Health Survey (SF-36) [38]. It measures self reported physical and mental health and has 36 items.

Attachment between the infant and the mother will be measured through the Maternal Postnatal Attachment Scale (MPAS) [39]. The scale comprises mothers’ emotional response to their infants and dimensions relating to mother-infant attachment and has 19 items.

Mothers satisfaction with breastfeeding will be measured through the Maternal Breastfeeding Evaluation Scale (MBFES) [40], which measures mothers’ satisfaction with breastfeeding and has 30 items.

Breastfeeding (i.e. exclusive, partial, none and method) and infant’s weight at six months of postnatal age will be measured through questions in the compiled questionnaire (instruments/scales presented above) sent to mothers.

**Table 2:** Time schedule for outcome measurements

**Measures**	**Baseline at discharge questionnaire**	**8 weeks after discharge phone call**	**8 weeks after discharge questionnaire**	**6 months PNA questionnaire**
Breastfeeding (exclusive, partial, non, method)	•	•	•	•
Breastfeeding satisfaction, MBFES (mother)			•	•
Parental stress, SPSQ (mother/partner)			•	•
Attachment, MPAS (mother)			•	•
Quality of life, SF-36 (mother/partner)	•		•	•
Infant’s health (well-being, visits to health care facilities (mother/partner)			•	•
Experience of BST telephone support (mothers/partners)			•	•
Experiences of breastfeeding support (at NICU and Child Health Service) (mothers/partners)			•	•

### Qualitative outcomes

Eight focus groups (four with participants from the intervention group and four from the control group) with about 8 mothers in each group will be held after the final follow up data from the study has been collected at 6 months, to avoid potential interactions. There will be an option to volunteer for qualitative interview on the questionnaire sent to the mothers 8 weeks after hospital discharge. This enables gaining rich information from women with a maximum diversity sample (i.e. age, SES, parity) for the focus groups. The focus of the group will be on experiences of the received support. Mothers who volunteer but are not selected to participate in a focus group will be given the opportunity to submit their comments through the study Website: http://www.amningsstod.se

In addition, after the study has finished, focus groups will be held with 1) the feeding support team (e.g. experiences of providing care during the trial, the opportunities and challenges, views about the workload and working relationships between the BST and other health service staff) and 2) staff working in the NICU who are not in the feeding teams (e.g. workload and work-organization in the unit) for each NICU, in total 8 groups.

### Process evaluation for the study

To evaluate intervention fidelity for person-centered telephone support throughout the study and between intervention and control groups, phone-calls made by each BST member on randomly selected dates, in the beginning, middle and at the end of the study period, will be recorded if the mother consents to it. Recorded phone calls will be analyzed with a thematic coding-scheme. This procedure is important in order to: 1) monitor that support given is person-centered and regardless of group (I or C) and to assess any change in implementation over time (beginning, middle or end of the study period).

In addition, during the study period, members of the research team will observe BST meetings (not when specific mothers are discussed) and NICU staff meetings. Furthermore, interviews with NICU managers will be conducted at the end of the study, with the aim to detect possible major changes (e.g. in care/staff-infant ratio/environment) or problems that have occurred during the study period. Observations and notes will be recorded in a reflective diary, which will contribute to the qualitative data analysis.

### Data analysis

All members of the research team will be blind to the group allocation (I, C) throughout the study period and during analyses of primary outcome. Intention-to-treat will be used. This means that analyses will include all randomized mothers in the groups to which they were randomly assigned, regardless of their adherence with the entry criteria, regardless of the treatment they actually received, and regardless of subsequent withdrawal from treatment or deviation from the protocol. To study the differences between intervention group and control group on the primary outcome measure; the proportion of mothers who exclusively breastfeed 8 weeks after discharge, a two-sided Chi - square test will used. The level of significance; p-value is set at p <0.05 in all analyses. The specific statistical analysis used with each outcome variable will be determined by the distribution of the outcome variables. Subgroup analyses will be conducted on SES (low vs. high), parity (primipara vs. multipara), and on GA (very preterm vs. preterm).

Secondary outcomes will potentially be analyzed with a mixed effect model. A mixed effect modeling approach has several advantages when conducting a multicenter study with repeated measurements compared to a conventional analysis. For example, a mixed model, offers an insightful analysis and improved precision by taking into account the different centers in the analysis. In addition, a mixed model does not need complete data from all subjects. The result from a mixed model analysis presents a more accurate estimation of the intervention and standard errors [41].

All qualitative interview data from focus groups with mothers will be recorded, transcribed and analyzed; responses to open questions in the questionnaire will be transcribed. Transcripts will be read by two researchers to independently identify categories and key themes according to qualitative content analysis [42]. The categories and key themes will be discussed at research team meetings and a final version will be agreed. All data from focus groups with staff will be analyzed as with mothers. In addition these will be informed by data from BST meeting and ward observations recorded in a reflective diary. Qualitative data collection and analyses will be performed rigorously and supervised by experienced qualitative researchers.

### Health economic evaluation

In the health economic analysis the incremental costs of proactive breastfeeding support will be compared to incremental benefits, in comparison to reactive breastfeeding support [43]. Three kinds of benefits will be considered in the analysis:

•Changes in breastfeeding rate will be linked to decreased risk of diseases and premature death, using best available data on risks of not breastfeeding. Less risk of disease will be transformed to gained quality adjusted life years (QALYs) for each disease’s impact on health care costs and production losses will also be considered.

•Parents’ quality of life, expressed in QALYs, will be measured until 6 months postnatal age, SF-6D based on SF-36 [38,44].

•Use of health care resources of the infant, during the follow up time (6 months) will be measured through questions of the infants’ health and wellbeing, illnesses, visits to health care facilities, apart from normal follow-up visits, in the questionnaire sent to mothers at 8 weeks after discharge and at 6 months PNA.

Costs are measured by the recorded number and duration of telephone calls in both study groups. Number and duration of calls are recorded by the BST in the log book. Cost-effectiveness will be analyzed using a cost-utility analysis. Cost-effectiveness ratio will be expressed as costs per gained QALY [43].

### Ethical considerations

The study has no obvious risks; in the pilot study mothers expressed their appreciation that somebody called. All eligible mothers and their partners who agree to participate in the study will sign a written consent. The mothers and the partners will be informed that participation in the study is voluntary and that they can withdraw at any time and that the questionnaires, answers and results are all anonymous and cannot be linked or influence the medical care they receive any way. The trial is conducted and monitored to minimize harm, if the mother wishes not to be called every day; the mothers have the option to decide when to be called. Log book, questionnaires, and recorded telephone calls are stored in a locked area, not accessible to unauthorized individuals. This study has been approved by the Regional Ethical Review Board, Uppsala 2012-08-15 Dnr: 2012/292.

## Discussion

This paper outlines a RCT designed for mothers of preterm infants to improve breastfeeding rates, mothers’ breastfeeding satisfaction and attachment, and parents’ health and wellbeing. It is designed to be feasible to implement in a NICU, in Sweden and internationally. Results from systematic reviews show that breastfeeding interventions may be more effective than usual care in increasing breastfeeding rates in both the short and long term [45,46]. A recommendation from systematic reviews of breastfeeding intervention is to measure exclusive breastfeeding in research [47]. It is also recommended to conduct an economic analysis to see the cost effectiveness of the intervention and in addition qualitative research to explore the nature of breastfeeding support and mechanisms in how support operates [46]. The intention-to-treat analyses strengthen the inferences from findings as we will include noncompliant mothers and will minimize potential bias. In the design, intervention and control groups will be able to access reactive support which is not standard practice. This strategy reduces the risk of the Hawthorne effect (motivational effect of the interest being shown in them) as all included mothers will be eligible to telephone the BST for support and outcome data will be measured identically for all groups. To reduce the risk for selection bias, very few exclusion criteria will be used, which strengthens the results and generalisability. A strength in this study is that data collection will take place in four hospitals in different counties, which will a) increase the scientific quality by reducing the risk of recruitment bias, b) facilitate participant recruitment within a feasible time, c) enable comparisons of both outcomes and intervention delivery process between different teams and NICU contexts which increases the generalisability to other settings. The process evaluation which combines qualitative interview, observation and call frequency data is carefully designed to illuminate any observed differences in outcomes between the four sites. Care has been taken to minimize both interactions between collection of process evaluation data and either the delivery of the intervention or collection of outcome data. A methodological challenge that may be relevant to this study is unexpected confounders. Although attention has been paid to potential confounders in the design, in which stratified block randomization is used to ensure comparability between groups, unexpected confounders may appear. This study provides a unique opportunity to determine if a proactive telephone support to breastfeeding mothers of preterm infants after discharge from NICU will improve breastfeeding rates, mothers’ breastfeeding satisfaction and attachment, and parents’ health and wellbeing.

## Abbreviations

BST: Breastfeeding Support Team; C: Control; CPAP: Continuous Positive Airway Pressure; GA: Gestational age; I: Intervention; MBFES: Maternal Breastfeeding Evaluation Scale; MPAS: Maternal Postnatal Attachment Scale; NEC: Necrotizing enterocolitis; NICU: Neonatal Intensive Care Unit; SES: Socioeconomic status; WHO: World Health Organization; PNA: Postnatal age; PSI: Parental Stress Index; QALY: Quality Adjusted Life Years; RCT: Randomized Controlled Trial; SF-36: Short Form health survey; SPSQ: Swedish Parental Stress Questionnaire.

## Competing interests

The authors declare that they have no competing interests.

## Authors’ contribution

JE, ME, LH, PH, RF were involved in the conception and in the design of the study. JE, RF were involved in the training of Breastfeeding Support Teams. JE drafted the manuscript with all authors contributing to the manuscript. All authors approved the final manuscript. RF, ME, LHW are involved with PhD supervision of JE on this project.

## Pre-publication history

The pre-publication history for this paper can be accessed here:

http://www.biomedcentral.com/1471-2431/13/73/prepub
